# Cryo-EM structures of PAC1 receptor reveal ligand binding mechanism

**DOI:** 10.1038/s41422-020-0280-2

**Published:** 2020-02-11

**Authors:** Jia Wang, Xianqiang Song, Dandan Zhang, Xiaoqing Chen, Xun Li, Yaping Sun, Cui Li, Yunpeng Song, Yao Ding, Ruobing Ren, Essa Hu Harrington, Liaoyuan A. Hu, Wenge Zhong, Cen Xu, Xin Huang, Hong-Wei Wang, Yingli Ma

**Affiliations:** 1 Beijing Advanced Innovation Center for Structural Biology, Tsinghua-Peking Joint Center for Life Sciences, School of Life Sciences, Tsinghua University, Beijing, 100084 China; 2 Amgen Asia R&D Center, Amgen Research, Bldg. 2, 13th Floor, No. 4560 Jinke Road, Shanghai, 201210 China; 3 School of Life and Health Sciences, Kobilka Institute of Innovative Drug Discovery, The Chinese University of Hong Kong, Tu H.L. Building (Research Building B) R705, Longxiang Road 2001, Longgang district, Shenzhen, 518172 Guangdong China; 4 Hybrid Modality Engineering, Therapeutic Discovery, Amgen Research, One Amgen Center Dr., Thousand Oaks, CA 91320 USA; 5 Department of Neuroscience, Amgen Research, One Amgen Center Dr., Thousand Oaks, CA 91320 USA; 6 Molecular Engineering, Therapeutic Discovery, Amgen Research, 360 Binney Street, Cambridge, MA 02142 USA

**Keywords:** Mechanisms of disease, Cryoelectron microscopy

## Abstract

The pituitary adenylate cyclase-activating polypeptide type I receptor (PAC1R) belongs to the secretin receptor family and is widely distributed in the central neural system and peripheral organs. Abnormal activation of the receptor mediates trigeminovascular activation and sensitization, which is highly related to migraine, making PAC1R a potential therapeutic target. Elucidation of PAC1R activation mechanism would benefit discovery of therapeutic drugs for neuronal disorders. PAC1R activity is governed by pituitary adenylate cyclase-activating polypeptide (PACAP), known as a major vasodilator neuropeptide, and maxadilan, a native peptide from the sand fly, which is also capable of activating the receptor with similar potency. These peptide ligands have divergent sequences yet initiate convergent PAC1R activity. It is of interest to understand the mechanism of PAC1R ligand recognition and receptor activity regulation through structural biology. Here we report two near-atomic resolution cryo-EM structures of PAC1R activated by PACAP38 or maxadilan, providing structural insights into two distinct ligand binding modes. The structures illustrate flexibility of the extracellular domain (ECD) for ligands with distinct conformations, where ECD accommodates ligands in different orientations while extracellular loop 1 (ECL1) protrudes to further anchor the ligand bound in the orthosteric site. By structure-guided molecular modeling and mutagenesis, we tested residues in the ligand-binding pockets and identified clusters of residues that are critical for receptor activity. The structures reported here for the first time elucidate the mechanism of specificity and flexibility of ligand recognition and binding for PAC1R, and provide insights toward the design of therapeutic molecules targeting PAC1R.

## Introduction

PACAP is a 38-amino acid C-terminally amidated polypeptide (PACAP38) that was discovered as a hypothalamic neuropeptide to potentially induce cAMP levels in anterior pituitary cells.^1^ The N-terminal 27 residues of PACAP38, highly conserved in almost all vertebrate species and responsible for the physiological activity of the peptide, undergo internal cleavage-amidation to generate the PACAP27 fragment. Because of the 68% sequence identity between PACAP27 and vasoactive intestinal polypeptide (VIP), PACAP is identified as a member of the glucagon/gastric inhibitory polypeptide (GIP)/secretin/VIP family—a hormone family consisting of evolutionarily related peptides that regulate the activity of class B G-protein coupled receptor (GPCR) family, also known as secretin receptor family.^2^

The receptors that recognize PACAP are characterized into three distinct subtypes based on their relative affinities to PACAP and VIP: the pituitary adenylate cyclase-activating polypeptide type I receptor (PAC1R) with two orders of magnitude higher affinity to PACAP than to VIP; the vasoactive intestinal polypeptide receptor 1 (VPACR1) and receptor 2 (VPACR2) with similar PACAP/VIP affinities.^3^

PACAP and its receptors are broadly expressed in the central nervous system (CNS) and in most peripheral organs, and have been found to exert a variety of functions including control of neurotransmitter release, vasodilation, bronchodilation, activation of intestinal motility, neuroprotection, immune modulation, and stimulation of cell proliferation and/or differentiation.^4^

As the major sensory and vasodilator neuropeptides, PACAP38 and VIP are involved in parasympathetic communication with the cranial vasculature. Abnormal activation and sensitization of the central trigeminovascular pain pathway mediate migraine and the release of these peptides.^5^ Intravenous infusion of PACAP38 but not VIP induces delayed migraine-like headaches, indicating that PAC1R is playing a major role in migraine^6^ and suggesting PACAP38-PAC1R as a potential therapeutic target for migraine treatment.^7^

Maxadilan, another natural PAC1R agonist, is a 61-amino acid polypeptide isolated from the salivary gland of the blood-feeding sand fly *Lutzomia lingipalpis*.^8^ Maxadilan is an immunomodulator and has been shown to facilitate the transmission and establishment of leishmaniasis. Despite the low sequence homology between maxadilan and PACAP38, they both potently activate PAC1R. The structural basis of receptor ligand recognition and activation mechanism remains unknown.

Recent development in cryo-electron microscopy (cryo-EM) has enabled the determination of full-length class B GPCR structures in complex with their peptide ligand and G protein complex.^9–13^ However, no structural information is available for PAC1R. Here we report the cryo-EM structures of G_s_-protein coupled PAC1R in complex with PACAP38 and maxadilan, respectively, and uncover the underlying mechanism of convergent activity from distinct ligands on a class B GPCR. Structure-guided mutagenesis elucidates the key residues responsible for ligand binding and receptor activation. These results further strengthen our mechanistic understanding of PAC1R regulation and will benefit future rational design of therapeutic molecules for migraine.

## Results

### Structure determination

For cryo-EM structure determination purpose, we modified the human PAC1R with a short C-terminal truncation (439–468), seven mutations (T163L, T167A, T169L and T170L on TM1; T276A, T278L and C280F on TM4), replacement of the native signal peptide by that of haemagglutinin (HA), and addition of affinity tags (an N-terminal FLAG tag and a C-terminal 10× His tag) (Supplementary information, Fig. S1). These modifications did not alter receptor ligand binding and pharmacological properties (Supplementary information, Fig. S2). We generated by site-directed mutagenesis the dominant-negative Gα_s_ construct, including mutations that reduce nucleotide affinity (S54N and G226A) and improve the dominant-negative effect (E268A, N271K, K274D, R280K, T284D, and I285T) to improve the complex stability.^10^

Human PAC1R, Gα_s_, Gβ_1_, and Gγ_2_ were co-expressed in High Five insect cells using baculovirus transfection to form the GPCR complex. Agonist peptide, PACAP38 or maxadilan, and Nanobody-35 (Nb35)^9,10^ were added during purification to enable a stable complex formation. The complex was solubilized in lauryl maltose neopentyl glycol (LMNG) and cholesteryl hemisuccinate, and then purified by nickel affinity and size exclusion chromatography to yield a monodisperse complex that contained all the components (Supplementary information, Fig. S2).

**Fig. 1:**
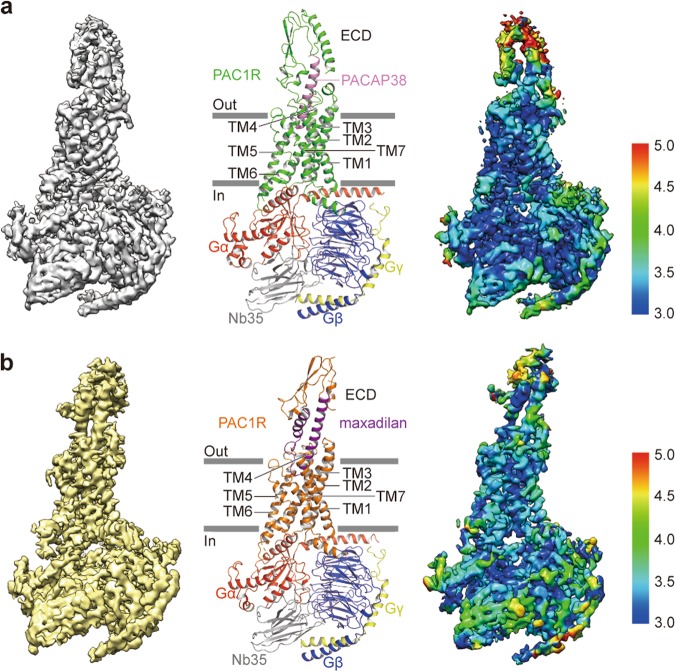
Cryo-EM structures of PACAP38-PAC1R-G_s_ and maxadilan-PAC1R-G_s_. **a, b** Cryo-EM density map (left), the structure model (middle) and local resolution distribution map (right) of PAC1R-Gs in complex with PACAP38 (**a**) and maxadilan (**b**). The structures of PAC1R (green in PACAP38-PAC1R-G_s_ structure, orange in maxadilan-PAC1R-G_s_ structure), PACAP38 (pink), maxadilan (purple), Gα (red), Gβ (blue), Gγ (yellow) and Nb35 (gray) are represented in cartoon.

Single-particle cryo-EM analysis of the complexes in vitreous ice yielded a final map at a resolution of 3.5 Å for PACAP38-PAC1R-G_s_ (reconstructed from 82,970 particles) and that of 3.6 Å for maxadilan-PAC1R-G_s_ (reconstructed from 58,451 particles) (Fig. 1; Supplementary information, Figs. S3, 4, Table S1). The density for the seven transmembrane helices (TMs), the ligands, and G protein complex are unambiguously determined based on the well-traced α-helices and aromatic side chains. The extracellular domain (ECD) is incomplete due to flexibility, but we were able to utilize the previously solved crystal structure^14^ to place it according to the partial density (Supplementary information, Fig. S5).

### Overall structures of the PAC1R-G_s_ complexes

PAC1R-G_s_ complexes, with PACAP38 or maxadilan bound at the extracellular side and G_s_ protein complex bound at the intracellular side of PAC1R, are in an active state (Fig. 1) with conformation reminiscent of the other class B GPCR-G_s_ complex structures.^9–13^ The overall reported resolution is 3.5 Å and 3.6 Å for the PACAP38-PAC1R-G_s_ and the maxadilan-PAC1R-G_s_ complexes, respectively (Fig. 1a, b). Although both structures represent G protein-bound active state of PAC1R, the ligands PACAP38 and maxadilan, with their dramatically different sequences and stereo structures, adopted disparate modes of binding to the ECD and the orthosteric site. To accommodate differences in ligand size, the TMs and extracellular loops (ECLs) that consist of the orthosteric site shift slightly with a root-mean-square deviation (r.m.s.d.) of 1.11 Å in the 7-TM region between the two structures.

### PACAP38-PAC1R binding interface

**Fig. 2:**
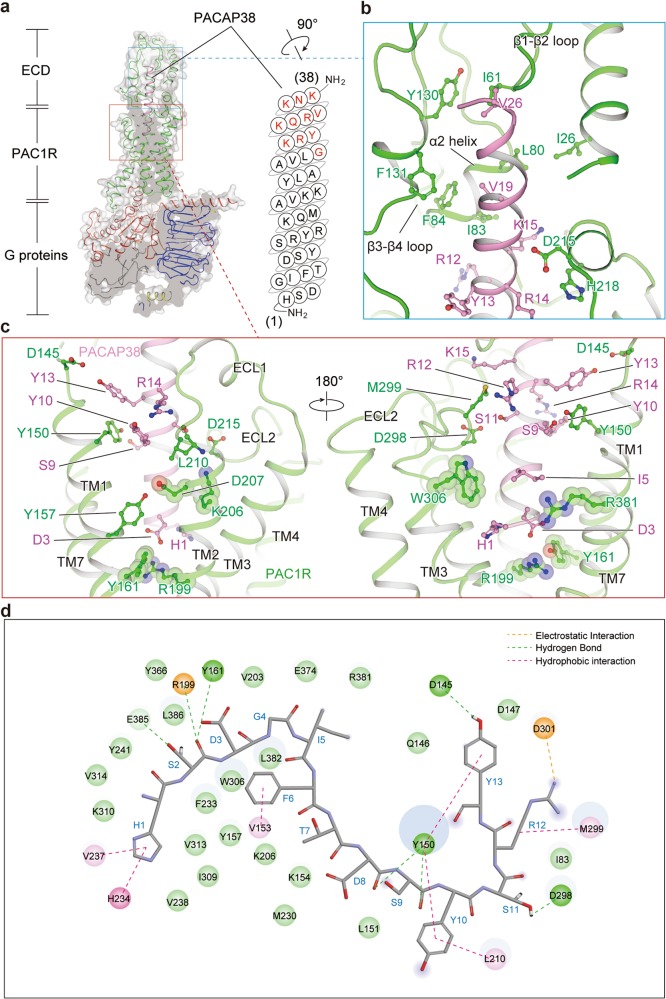
PACAP38 ligand binding site. **a** Slice view of the PACAP38-PAC1R-G_s_ structure and a schematic helical sequence for PACAP38 with unresolved residues in red. **b** Close view of the C-terminal part of PACAP38 showing detailed interactions. **c** Close views of the N-terminal part of PACAP38 from two angles with 180° rotation. The effective residues in mutagenesis assay are presented by spheres. **d** The PAC1R-PACAP38 interaction diagrams. PACAP38 (H1-Y13) is shown as sticks. Residues are represented as spheres and colored by interaction type. Interactions between the residues and the ligand atoms are drawn as dashed lines, colored by interaction type. The solvent accessible surface of an interacting residue and of an atom are represented by haloes around the residue and the atom. The diameter of the circle is proportional to the solvent accessible surface.

The PAC1R construct used in the cryo-EM study retains ligand binding and pharmacological properties comparable to the wild-type (WT) receptor (Supplementary information, Fig. S2). The peptide forms an α-helix with its N-terminus inserted into the orthosteric site. Structure of the N-terminal 27 residues, conserved and responsible for receptor activation, are unambiguously resolved^15^ (Fig. 2a), while the C-terminal 11 residues are not visible in the map probably due to flexibility and omitted from the final reported structure.

Outside the orthosteric site, PACAP38 mainly forms hydrophobic interactions with the receptor in the ECD region (residues 53–88) that is previously reported to be critical for ligand binding affinity^16^: K15_P (PACAP38)_ and V19_P_ hydrophobically interact with I83^ECD^ and F84^ECD^ of the β3–β4 loop; V26_P_ forms hydrophobic alkyl interaction with I61^ECD^ of the β1–β2 loop (Fig. 2b).

Within the orthosteric site, PACAP38 interacts with PAC1R through TM1, TM2 and ECL1 on one side and TM3 and ECL2 on the other side. On TM1, Y150^1.36^ forms hydrogen bond with S9_P_ and hydrophobic interaction with Y10_p_ and Y13_P_. D145^1.31^ forms hydrogen bond with Y13_P_. On TM2, PAC1R L210^2.71^ forms hydrophobic interactions with Y10_P_ and establishes hydrogen bond with R14 via its main chain. ECL1 closely attaches to PACAP38, but under current resolution we cannot resolve specific residue interactions (Fig. 2c). On the other side, D298^ECL2^ forms hydrogen bond with S11_p_. M299^ECL2^ interacts with R12_P_ and K15_P_ hydrophobically_._ In addition, W306^5.39^ may form hydrophobic interactions with H1_P_ and I5_P_. At the bottom of the orthosteric site, Y161^1.47^ and R199^2.60^ form hydrogen bonds with D3_P_ (Fig. 2c, d; Supplementary information, Table S2).

### Maxadilan-PAC1R binding interface

**Fig. 3:**
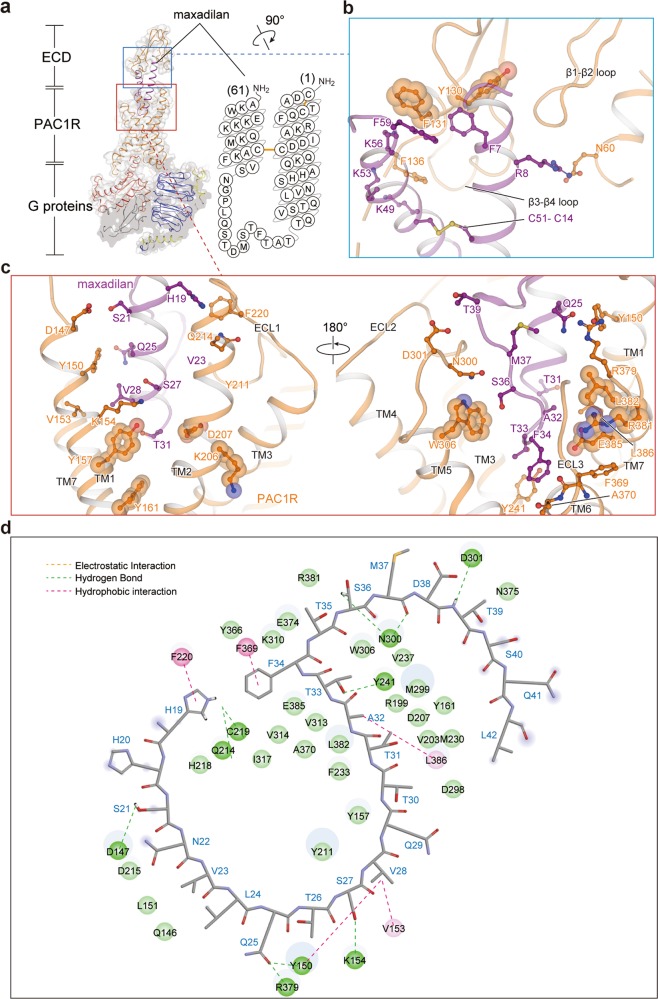
Maxadilan orthosteric site. **a** Slice view of the maxadilan-PAC1R-G_s_ structure and a schematic helix-loop-helix sequence for maxadilan. **b** Close view of maxadilan from outside of the binding pocket showing detailed interactions. **c** Close views of the insides of the orthosteric site from both sides with 180° rotation. The effective residues in mutagenesis assay are presented by spheres. **d** The PAC1R-Maxadilan interaction diagrams. Maxadilan (H19-L42) is shown as sticks. Residues are represented as spheres and colored by interaction type. Interactions between the residues and the ligand atoms are drawn as dashed lines, colored by interaction type. The solvent accessible surface of an interacting residue and of an atom are represented by haloes around the residue and the atom. The diameter of the circle is proportional to the solvent accessible surface.

Maxadilan is a similar ligand compared to PACAP38 in terms of ligand affinity and receptor activation, however, the structure is very different (Supplementary information, Fig. S2). Maxadilan forms the N- and C-terminal helices that are linked by a loop, deletion of which converts the peptide into an antagonist (Fig. 3a).^17^ In model building, we defined the residues of the helices based on the disulfide bond between C14_M (maxadilan)_ and C51_M_,^18^ as well as the side chain densities of aromatic residues (Fig. 3a). In the maxadilan-PAC1R-G_s_ structure, PAC1R ECD adopts a dramatically different conformation from that of the PACAP38-PAC1R-G_s_ structure, but intriguingly utilize the same interface for maxadilan binding. Some detailed interactions are not well defined because of the incomplete ECD map; the map for aromatic residues such as F7_M_ and F59_M_, and Y130^ECD^, F131^ECD^ and F136^ECD^ on the α3 helix is of great quality and reveals hydrophobic interaction network among these residues (Fig. 3b).

Within the orthosteric site, maxadilan, bearing a much larger size and a distinct shape in contrast to PACAP38, establishes different interactions with the receptor. On one side, PAC1R closely interacts with the C-terminal helix of maxadilan through TM1 and ECL1. On TM1, D147^1.33^, Y150^1.36^ and K154^1.40^ form hydrogen bonds with S21_M_, Q25_M_ and S27_M_, respectively. Y150^1.36^ and V153^1.39^ interact with V28_M_ through hydrophobic interactions. On ECL1, Q214^ECL1^ backbone forms hydrogen bond with H19_M_ whose side chain hydrophobically interacts with F220^ECL1^ (Fig. 3c). On the other side of maxadilan, the loop wedges into the orthosteric site to interact with ECL2, TM6 and TM7. On ECL2, N300^ECL2^ interacts with S36_M_ and M37_M_ (backbone) hydrophilically, and D301^ECL2^ forms hydrogen bonds with T39_M_. On TM6, the backbones of F369^6.56^ and A370^6.57^ stabilize F34^M^ through amide-Pi stacked interaction. On TM7, R379^ECL3^ forms hydrogen bonds with Q25_M_ and L386^7.43^ hydrophobically interacts with A32_M_ (Fig. 3c, d; Supplementary information, Table S2).

### PAC1R activation and G protein engagement

Peptide ligands associate with the large ECD of class B GPCRs and interact with the transmembrane bundles to activate the receptors. The interaction induces reorganization of the buried polar residues in the TM bundle, which is believed to reflect the mode of receptor activation.^19–21^ Three layers of highly conserved polar networks governed this process: (i) on the top is the central polar network formed by residues R^2.60^, N^3.43^, H^6.52^ and Q^7.49^,^19,21^ (ii) in the middle is the HETX polar networks formed by H^2.50^, E^3.50^, T^6.42^, Y^7.57^, (iii) at the bottom is the TM2-6-7-8 network formed by R^2.46^, R^6.37^, N^8.47^, E^8.49^ (Supplementary information, Fig. S6).^20^ Similar to other class B GPCRs, dissociation of the central hydrogen bond network upon peptide binding induces destabilization of TM6 around the P^6.47^XXG^6.50^ motif, which enables further rearrangement in the lower polar networks to facilitate receptor activation and G protein engagement. Upon dissociation of the HETX polar network, the side chain of Y400^7.57^ in both PAC1R structures shifts away and T355^6.42^ further moves away along with the bended TM6 (Supplementary information, Fig. S6). Mutations at T355^6.42^ to destabilize the HETX network and at the P^6.47^XXG^6.50^ motif to stabilize the active conformation both result in constitutive activation of PAC1R (data not shown).

Upon peptide ligand-induced activation, PAC1R engages G_s_ heterotrimeric protein to the transmembrane bundle on the cytoplasmic side with a cavity, formed by outward bending of TM6, to interact with the G_αs_ Ras-like domain. This extensive interface consists of both hydrophobic and electrostatic interactions in a similar pattern as reported in other G_αs_-class B GPCR complexes.^9,10^ These interactions are summarized in Supplementary information, Fig. S7 and Table S3. We report more interactions from the PACAP38-PAC1R-G_s_ mainly due to the better map quality of this structure. A key difference in the activation of class B GPCRs comes from the additional hydrophilic interactions formed between helix 8 (H8) and G_β_ protein, compared to class A GPCRs.^11^ In PAC1R, we observed this conserved interaction between R413^8.56^, K180^ICL1^ and H311, D312 on G_β_, respectively (Supplementary information, Fig. S7 and Table S3). Like other class B GPCRs in active conformation, H8 interacts with detergent micelle with bulky aromatic residues on the membrane-proximal face (Supplementary information, Fig. S7).^9,10^

### PAC1R ligand binding specificity and flexibility

To identify critical residues responsible for ligand-induced receptor activation, we conducted virtual alanine scanning on all the residues within the ligand-binding pockets and evaluated the destabilizing effects through mutation energy analysis (Supplementary information, Fig. S8). We then selected the most effective residues on the mutation energy ordering (> 0.5 kcal/mol) and key interacting residues derived from structural analysis, designed single point alanine substitutions and tested their effects on PAC1R activation experimentally (Supplementary information, Table S4).

Y130^ECD^A and F131^ECD^A showed significant decreases in EC_50_ of maxadilan- but not PACAP38-induced receptor activity in the functional cAMP assay, and both mutations, particularly F131A, failed to compete for I^125^-PACAP27 binding in the competition binding assay, indicating that the hydrophobic interactions are crucial for binding of maxadilan to induce receptor activation (Fig. 3b; Supplementary information, Table S4 and Fig. S9). Y157^1.43^A showed decrease of EC_50_ of maxadilan-induced receptor activity, consistent with the maxadilan-PAC1R-G_s_ structure where Y157^1.43^ is close to the loop region of maxadilan (Fig. 3c). Intriguingly, the alanine substitution of R199^2.60^ specifically affected PACAP38-induced receptor activity and absolutely lost binding affinity to I^125^-PACAP27 (Supplementary information, Table S4 and Fig. S9). It is consistent with the structural observation that PACAP38 binds much deeper than maxadilan and establishes additional interaction through hydrogen bond with D3_P_ (Fig. 2c; Supplementary information, Table S2). Y161^1.47^, with side chain pointing to the same position as R199^2.60^, showed similar preference of PACAP38 function (Fig. 2c; Supplementary information, Table S2). Both indicate that PACAP38 requires additional interactions on its N-terminus for ligand binding and function. In GLP1 receptor, K197^2.67^ and D198^2.68^ hold the aromatic moiety of Y148^1.43^ in an optimal position for ligand binding and receptor activity.^22^ Similarly, alanine substitutions of the corresponding residues K206^2.67^ and D207^2.68^ in PAC1R lost receptor activity with both ligands (Supplementary information, Table S4). In GLP1 receptor, mutation of W306^5.36^ to alanine results in reduced potency by > 200-fold.^22–24^ Similarly, in PAC1R W306^5.36^A led to significant decrease of activities to both ligands (Supplementary information, Table S4).

The density map of M299^ECL2^ and D301^ECL2^ did not allow unambiguous fitting of side chain and interpreted direct interactions with maxadilan, but alanine substitution of either M299 or D301 specifically disturbed maxadilan-induced receptor activation. It might alter the conformation of ECL2 that is an important region for peptide ligand recognition.^23,24^ L382^7.39^A and E385^7.42^A significantly and specifically decreased the potency of maxadilan-induced receptor activation, but no structural relevance was observed from the structure (Supplementary information, Table S2). They might alter the binding pocket, thus specifically disturbing the mode by which maxadilan induced receptor activation.

**Fig. 4:**
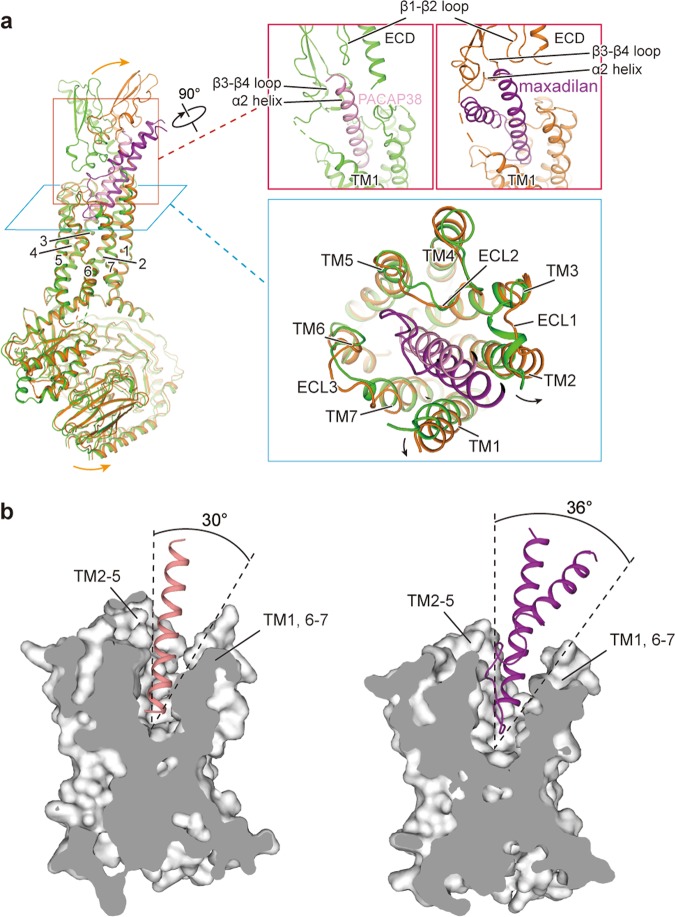
Comparison of PACAP38-PAC1R-G_s_ and maxadilan-PAC1R-G_s_ structures. **a** Superposition of PACAP38-PAC1R-G_s_ (green cartoon) and maxadilan-PAC1R-G_s_ (orange cartoon) structures. Close views from outside of the cell membrane (red squares) showing the PACAP38 and maxadilan binding in the orthosteric site. Close view (blue square) showing that the TM1 and TM2 shift outward in maxadilan binding mode. **b** Slice side views of the receptor, in parallel to the cell membrane, showing the binding pocket for PACAP38 (pink) and maxadilan (purple). The angels of the ‘cleft’ between TM2–5 and TM1, 6–7 are shown by dash lines.

PAC1R ECD displays dramatic conformational flexibility to accommodate two stereo distinct ligands, PACAP38 and maxadilan. Both peptides utilize the same ECD interface, consisting of the β1–β2 loop, the β3–β4 loop, and the α2 helix, for binding, resulting in different PAC1R ECD orientations (Fig. 4a). In both ligand binding modes, the TM1-ECD loop half embraces the peptide blocking it from escaping once the ligand inserts into the orthosteric site. These structural features demonstrate a ligand recognition and binding mechanism where ECD fits the divergent peptide orientation with flexible conformation and holds the peptide in the orthosteric site with steric hindrance (Fig. 4a). In line with the size and binding orientation of ligands, the ligand-binding pocket consisting of the upper parts of TMs and ECLs was also slightly reorganized. TM6, ECL3, and TM7 slightly shift outward in the PACAP38 structure, whereas TM1, ECL1 and TM2 in the maxadilan structure outward shift to fit for the extended conformation of maxadilan N-terminal helix (Fig. 4a). Maxadilan wedged the ligand-binding pocket more open by ~6° (Fig. 4b), demonstrating the flexibility for PAC1R ligand recognition.

## Discussion

**Fig. 5:**
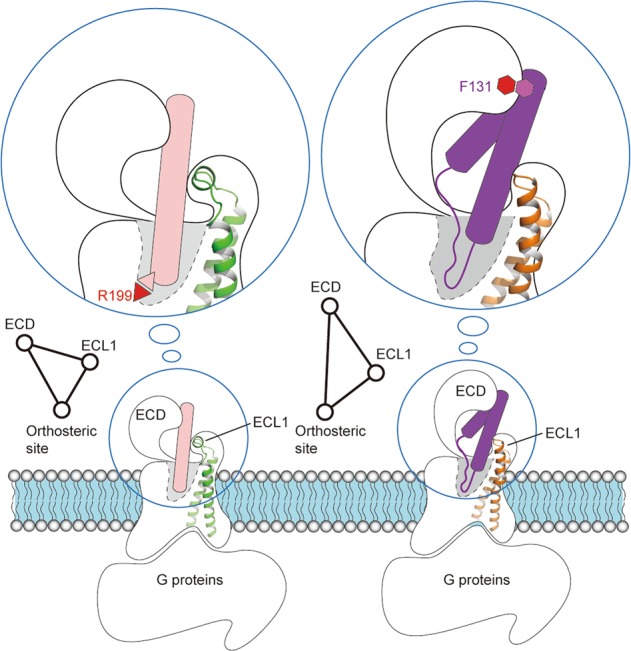
Schematic diagram of two ligand binding modes of PAC1R. Close views showing that ECD and ECL1 in different orientations ‘holding’ the peptides are highlighted in blue circles. The helices in PACAP38 and maxadilan are shown as pink and purple rods. The conformations of ECL1 in two structures are shown in cartoon. The relative positions of ECD and ECL1 are presented in circles and lines, revealing the flexibilities for different ligand binding. The hydrophilic interaction for PACAP38 is represented by triangle and the hydrophobic interaction for maxadilan is represented by hexagon.

PAC1R recognizes and binds the ligand as a ‘glove’, with the ‘palm’ (ECD) and the ‘thumb’ (ECL1) holding the ligand in the orthosteric site through hydrophilic and hydrophobic interactions (Fig. 5). ECD is flexible to fit for different orientations of ligand and connects to TM1 with a long loop which embraces the ligand and keeps it from escaping. ECL1 interacting with the ligand on the other side further stabilizes ligand binding (Fig. 5). The holding conformation of ECD and ECL1 enables PACAP38 or maxadilan to insert into the orthosteric site and form similar interactions for receptor activation. This ‘glove’ ligand binding mechanism is reminiscent of the two-domain binding hypothesis proposed for PAC1R,^25^ where the ECD creates a locally high concentration of ligand through high-affinity interactions with the ECD binding part of the ligand and further facilitates the orthosteric binding part to insert into the orthosteric site.

Although PACAP38 and maxadilan activate the PAC1R in similar potencies and induce similar conformational changes for G protein engagement, there are subtle differences in ligand binding and receptor activation reflected by the structures and functional assays. PACAP38 requires R199^2.60^ at the bottom of the pocket for binding to the pocket and could use this interaction to affect the central polar network for receptor activation. As for maxadilan, the loop does not insert into the pocket as deep as PACAP38 to reach R199^2.60^, whereas the interface between the loop and TM7 is likely an important region for receptor activation as confirmed by mutagenesis of L382^7.39^A and E385^7.42^A. These structural insights explain the previously reported results that maxadilan, with Q25–Q41 deleted, was an antagonist. This deleted form of maxadilan will not be able to make interactions with critical residues on TM7 or insert into the orthosteric site, hence fail to activate the downstream elaborate polar networks.^17^ In contrast to PACAP38, maxadilan is unlikely to rely on the residues within the pocket for ligand binding but relies on binding to the ECD through hydrophobic interaction with F131^ECD^. These observed subtle differences in ligand binding will be useful for fine-tuning the affinity and activity of PACAP38 and maxadilan for therapeutic purposes.

We compared PACAP38-PAC1R-G_s_ structure to previously solved structures of class B GPCRs bound with single-helix peptide ligand to try to understand the complex sequence-structure relationship. Sequence alignment indicates that GLP1 shows some similarity to PACAP38, whereas PTH shows barely any sequence resemblance to PACAP38 (Supplementary information, Fig. S10a). However, structure wise, PTH-PTH1R-G_s_ has a more similar peptide binding orientation and the ECD and ECL1 conformations are almost identical to PACAP38-PAC1R-G_s_, whereas in the GLP1-GLP1R-G_s_ structure, GLP1 peptide slightly swings away and the ECD and ECL1 conformations are dramatically different (Supplementary information, Fig. S10b). CGRP, with unstructured loops in both N- and C- terminal regions, is totally different from PACAP38-PAC1R-G_s_ by both sequence and structural alignments (Supplementary information, Fig. S10b).

## Conclusion

Delineating the properties of ligand binding specificity and flexibility of PAC1R may guide rational design of novel molecules with improved specificity and desired therapeutic effects. We report cryo-EM structures of PACAP38-PAC1R-G_s_ and maxadilan-PAC1R-G_s_ complexes, revealing two distinct PAC1R ligand binding modes. These structures reveal the conformational plasticity of ECD to recognize distinct ligands, and the specificity and flexibility of PAC1R ligand binding site to accommodate different ligands towards inducing convergent receptor activity in different modes. By molecular modeling, structure-guided mutagenesis and functional assay, we characterized and identified critical residues for ligand-induced receptor activation. These results provide the structural basis for PAC1R ligand recognition and bring insights into the receptor activation mechanism.

## Materials and methods

### Constructs

The human PAC1R was modified with an N-terminal deletion (89–109) that is a PAC1R splice variant with similar affinities to PACAP.^16^ A short C-terminal truncation (439–468) and seven mutations, T163L, T167A, T169L, T170L, T276A, T278L, C280F were introduced to increase protein yield and stability in micelle. The construct was cloned into both mammalian and insect cell expression vectors with replacement of the native signal peptide by that of haemagglutinin (HA) and addition of affinity tags (an N-terminal FLAG tag epitope and a C-terminal 10× His tag). These modifications did not alter receptor pharmacology (Supplementary information, Fig. S2). A dominant-negative Gα_s_ (DNGα_s_) construct was generated by site-directed mutagenesis to incorporate mutations that reduce nucleotide affinity (S54N and G226A) and improve the dominant-negative effect (E268A, N271K, K274D, R280K, T284D, and I285T). The DNGα_s_ was reported to have enhanced interaction between Gα and Gβγ subunits and improved overall trimeric complex stability.^10^ The 8× His-tagged human Gβ_1_ and human Gγ_2_ were cloned into pFastBac-dual vector.

### Expression

Human PAC1R, human DNGαs, and 8× His-tagged human Gβ_1_ and human Gγ_2_ were expressed in High Five insect cells using baculovirus. Cell cultures were grown in ESF921 serum-free medium to a density of 2.5 × 10^6^ viable cells/mL and then infected with three separate baculoviruses at a ratio of 1:1:1 for PAC1R, DNGα_s_, and Gβ_1_γ_2_. The culture was collected by centrifugation 48 h after infection and cell pellets were stored at –80 °C. Nanobody-35 (Nb35) was expressed in the periplasm of *Escherichia* *coli* strain WK6, extracted, and purified by nickel affinity chromatography according to previously described methods.^26^

WT PAC1R and mutations used in the functional assay were cloned into pJIF1.1 for BacMam virus generation.

### Complex purification

The cell pellet was thawed in 20 mM HEPES, pH 7.5, 100 mM NaCl, 2 mM MgCl_2_ supplemented with cOmplete Protein Inhibitor Cocktail tablets (Roche). Complex formation was initiated by addition of 10 μM PACAP38, Apyrase (25 mU/mL, NEB) and Nb35-His (10 μg/mL), the suspension was incubated for 1 h at 4 °C. Complexes from membranes were solubilized by 1% (w/v) lauryl maltose neopentyl glycol (L-MNG, Anatrace) complemented with 2 mM cholesteryl hemisuccinates (CHS, Anatrace) for 2 h at 4 °C. Insoluble material was removed by centrifugation at 40,000 rpm for 30 min and the solubilized complex was immobilized by batch binding to Ni-NTA resin (Qiagen). The resin was packed into a disposable plastic column (Bio-Rad) and washed with 20 column volumes of 20 mM HEPES, pH 7.5, 100 mM NaCl, 2 mM MgCl_2_, 0.01% (w/v) L-MNG, 20 μM CHS, and 50 mM Imidazole, and eluted with 4 column volumes of 20 mM HEPES, pH 7.5, 100 mM NaCl, 2 mM MgCl_2_, 0.01% (w/v) L-MNG, 20 μM CHS, and 300 mM Imidazole. The PAC1R-DNGs-Nb35 complex was then concentrated using an Amicon Ultra Centrifugal Filter (MWCO 100 kDa) before being subjected to size exclusion chromatography on a Superose 6 Increase 10/300 column (GE Healthcare) pre-equilibrated with 20 mM HEPES, pH 7.5, 100 mM NaCl, 2 mM MgCl_2_, 0.01% (w/v) L-MNG and 20 μM CHS to separate complex from contaminants. Eluted fractions consisting of monomeric receptor and G-protein complex were pooled and concentrated for electron microscopy experiments. The final yield of the purified complex was ~1 mg/L from insect cell culture.

Samples collected from each purification step were analyzed by SDS-PAGE. Precast gradient TGX gels (Bio-Rad) were used and stained by SimplyBlue (Invitrogen).

### Cryo-EM sample preparation and data collection

EM grids (Quantifoil, 300 mesh golden R1.2/1.3) were glow discharged for 20 s using Harrick plasma cleaner (Harrick). Vitrified specimen was prepared by applying 3.5 μL of 5 mg/mL protein complex solution on the grid in the Vitrobot chamber (FEI Vitrobot Mark IV) with blotting time of 3 s. The chamber of Vitrobot was set to 100% humidity at 18 °C. Cryo-EM data were collected on a Titan Krios electron microscope operated at 300 kV equipped with a Gatan K2 Summit direct electron detection camera (Gatan) using AutoEMation.^27^ Micrographs were recorded in super-resolution mode at a nominal magnification of 105,000×, resulting in a physical pixel size of 0.5455 Å per pixel. Defocus values varied from –1.5 μm to –2.5 μm. The dose rate was 8.0 electron per pixel per second. Exposures of 5.6 s were dose-fractionated into 32 sub-frames, leading to a total accumulated dose of 50 electrons per Å^2^ on the specimen.

### Image processing and 3D reconstruction

The raw super-resolution dose-fractionated image stacks were 2× Fourier binned, aligned, dose-weighted and summed using MotionCorr2.^28^ Contrast transfer function (CTF) parameters were estimated using CTFFIND4.^29^ Bad micrographs were removed manually based on the CTF parameters. The following processing steps were performed in RELION.^30^ For all the datasets, manually picked sets of particles were subjected to 2D classification. These generated templates for reference-based particle picking, respectively. Several rounds of reference-free 2D classification were performed to remove contaminants and bad particles from the automatically picked particle datasets. Initial models were generated using the “3D initial model” panel in RELION-3.0. After several rounds of reference-based 3D classification, the most homogeneous particles were selected for the final 3D auto-refinement. Local resolution distribution was estimated using “blocres” command in BSOFT software package.^31^ More details related to data processing are summarized in Supplementary information, Figs. S3, 4, Table S1.

### Model building and refinement

An initial model was generated by homology modeling using the GLP1-GLP1R-G_s_ cryo-EM structure (PDB: 5VAI), and the ECD model also refers to the isolated GLP1R ECD structure (PDB: 3IOL) and PAC1R ECD structure (PDB: 3N94). Manual building and adjustment were performed in Coot.^32^ DNGα, Gβ, Gγ and NB35 models were taken from ExP5-GLP1R-G_s_ structure (PDB: 6B3J). The ECD of maxadilan-PAC1R-G_s_ (PDB: 3N94) was rigid-body docked into the model with manual adjustment owing to limited density. The final models were subjected to real space refinement and minimization using PHENIX.^33^ Model validation was performed using MolProbity.^34^

### Molecular modeling

The structure preparation and virtual mutation calculation were performed with Discovery Studio (Dassault Systèmes BIOVIA, Discovery Studio Modeling Environment, Release 2017, San Diego: Dassault Systèmes, 2016). The PACAP38-PAC1R-G_s_ and the maxadilan-PAC1R-G_s_ complex structures were protonated at pH 7.4 with “Prepare Protein” protocol. Then the interactions between PAC1R and peptide ligands were analyzed with “Analyze Protein Interface” protocol. The identified interface residues were virtually mutated to Ala and for every single mutant, the differences in the free energy of binding between the WT and mutated structures are calculated with “Calculate Mutation Energy (Binding)” protocol. The “Effect of Mutation” is defined by default setting: Stabilizing, mutation energy is less than –0.5 kcal/mol; Neutral, mutation energy is between –0.5 and 0.5 kcal/mol; Destabilizing, mutation energy is greater than 0.5 kcal/mol.

The receptor-ligand interaction diagrams were generated for part of the ligands (PACAP38: H1-Y13; maxadilan: H19-L42) and PAC1R residues at the binding interface, with “Draw 2D Ligand Interaction Diagram” tool in Discovery Studio.

### Transient expression by BacMam virus

CHO-K1 cells were cultured in DMEM supplemented with 10% FBS and infected with BacMam viruses with a MOI of 100. 24 h after infection, cells were collected for membrane preparation or functional assay.

### Functional cAMP assay

Activation of WT PAC1R and mutants was measured based on intracellular cAMP levels using Lance Ultra cAMP kit (PerkinElmer) according to the manufacturer’s protocol. Briefly, in a well of 384-well plate, 1000 cells in 5 μL assay buffer (HBSS buffer pH 7.4 with 5 mM HEPES, 0.1% BSA and 0.5 mM IBMX) were mixed with 5 μL of different concentrations of PACAP38 or Maxadilan in assay buffer, incubated for 30 min at 37 °C. Then 5 µL of 4× Eu-cAMP and 5 µL of 4× Ulight-Anti-cAMP working solutions were added to each well and incubated at room temperature for 60 min before reading with Envision (PerkinElmer). All signals (ratio of 665 nm/615 nm) were fit with a sigmoidal dose-response model using GraphPad Prism 7.

### Membrane preparation and radioligand binding assay

CHO-K1 cells were infected with BacMam virus using method mentioned above. Cells were then pelleted and membrane was generated following the protocol by Ban et al.^35^ In radioligand saturation binding assay, membrane of WT PAC1R or PAC1R mutants was incubated with a dose concentration of ^125^I-PACAP27, starting from 1 nM, in binding buffer (50 mM HEPES, pH 7.4, 5 mM MgCl_2_, 1 mM CaCl_2_, 0.2% BSA). In radioligand competition binding assay, membrane of WT PAC1R or PAC1R mutants was incubated with a dose concentration of PACAP38 or Maxadilan in binding buffer containing 0.2 nM ^125^I-PACAP27. Binding reaction system was incubated at 30 °C for 2 h with gentle shaking, then filtered in UniFilter GF/B filtration Plate (PEI Coated, PerkinElmer), and washed three times immediately with ice-cold washing buffer (50 mM HEPES, pH 7.4, 500 mM NaCl, 0.1% BSA) using FilterMate™ Universal Harvester (PerkinElmer). After the plates were dried at 37 °C for 2 h, scintillation cocktail was added to each well, and radioactivity was counted in MicroBeta Trilux (PerkinElmer). Non-specific binding was determined in the presence of 100 nM PACAP38. All data were fit with one-site binding model using GraphPad Prism 7.

## Supplementary information

Supplementary information, Fig. S1

Supplementary information, Fig. S2

Supplementary information, Fig. S3

Supplementary information, Fig. S4

Supplementary information, Fig. S5

Supplementary information, Fig. S6

Supplementary information, Fig. S7

Supplementary information, Fig. S8

Supplementary information, Fig. S9

Supplementary information, Fig. S10

Supplementary information, Table S1

Supplementary information, Table S2

Supplementary information, Table S3

Supplementary information, Table S4
