# Supplementary material for: Cryo-EM structures of PAC1 receptor reveal ligand binding mechanism
Source: Cell Res. 2020 Feb 11;30(5):436–45. doi: 10.1038/s41422-020-0280-2 (PMC7196072; doi:10.1038/s41422-020-0280-2)
Supplement: Supplementary file 1 — Supplementary information, Fig. S1 [file 41422_2020_280_MOESM1_ESM.pdf]

Supplementary information, Figure S1

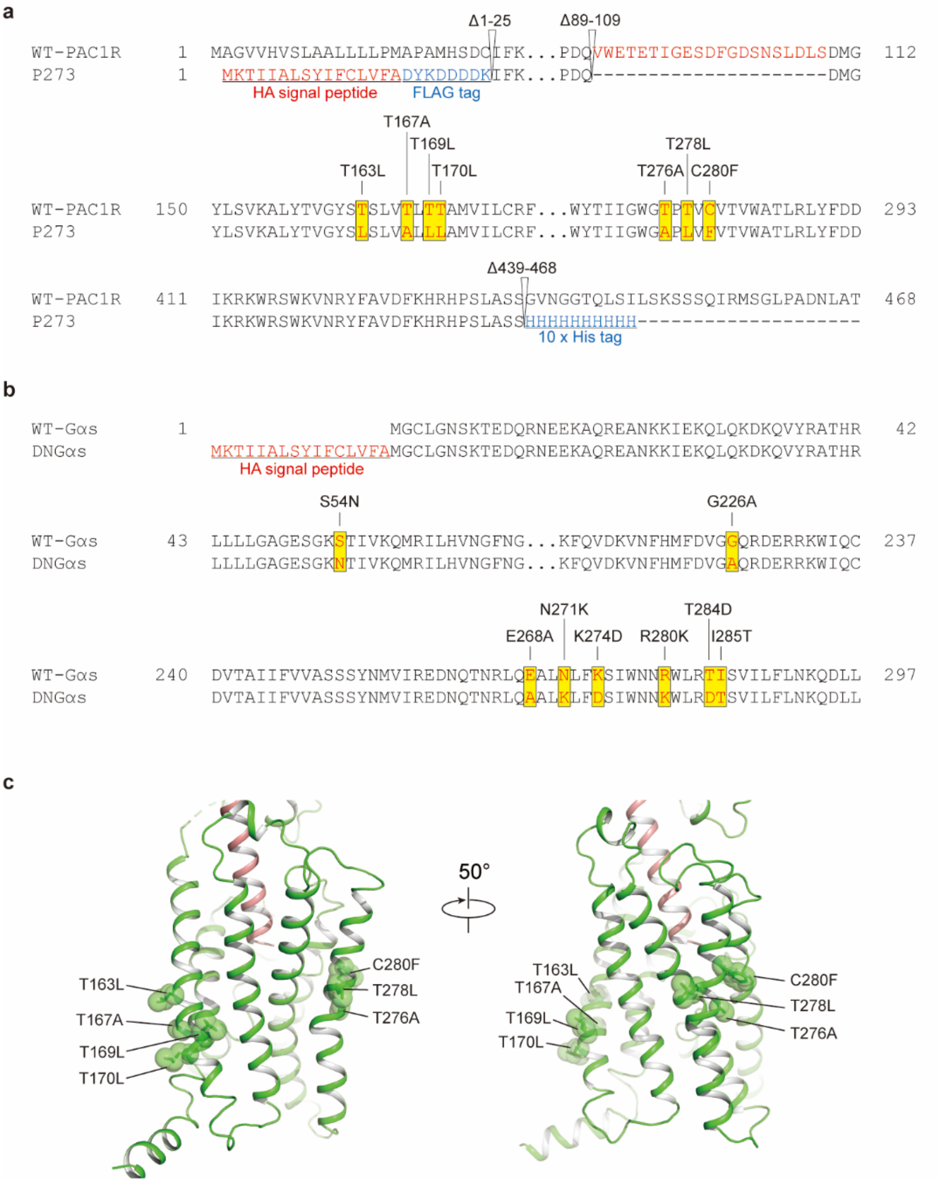

Fig. S1 Amino acid sequences of PAC1R, Gα_s constructs used for structure determination. **a, b** PAC1R (**a**) and Gα_s (**b**) are compared with the wild-type sequences to highlight mutations and modifications. Locations of mutated sites are highlighted in yellow. Deletions are shown by triangles. Sequence modifications are shown in red and blue. **c** Structural locations of the thermostability mutations introduced in the PAC1R. The 7 mutations, shown in spheres, are away from the ligand-binding pocket.
