# Supplementary material for: Cryo-EM structures of PAC1 receptor reveal ligand binding mechanism
Source: Cell Res. 2020 Feb 11;30(5):436–45. doi: 10.1038/s41422-020-0280-2 (PMC7196072; doi:10.1038/s41422-020-0280-2)
Supplement: Supplementary file 2 — Supplementary information, Fig. S2 [file 41422_2020_280_MOESM2_ESM.pdf]

Supplementary information, Figure S2

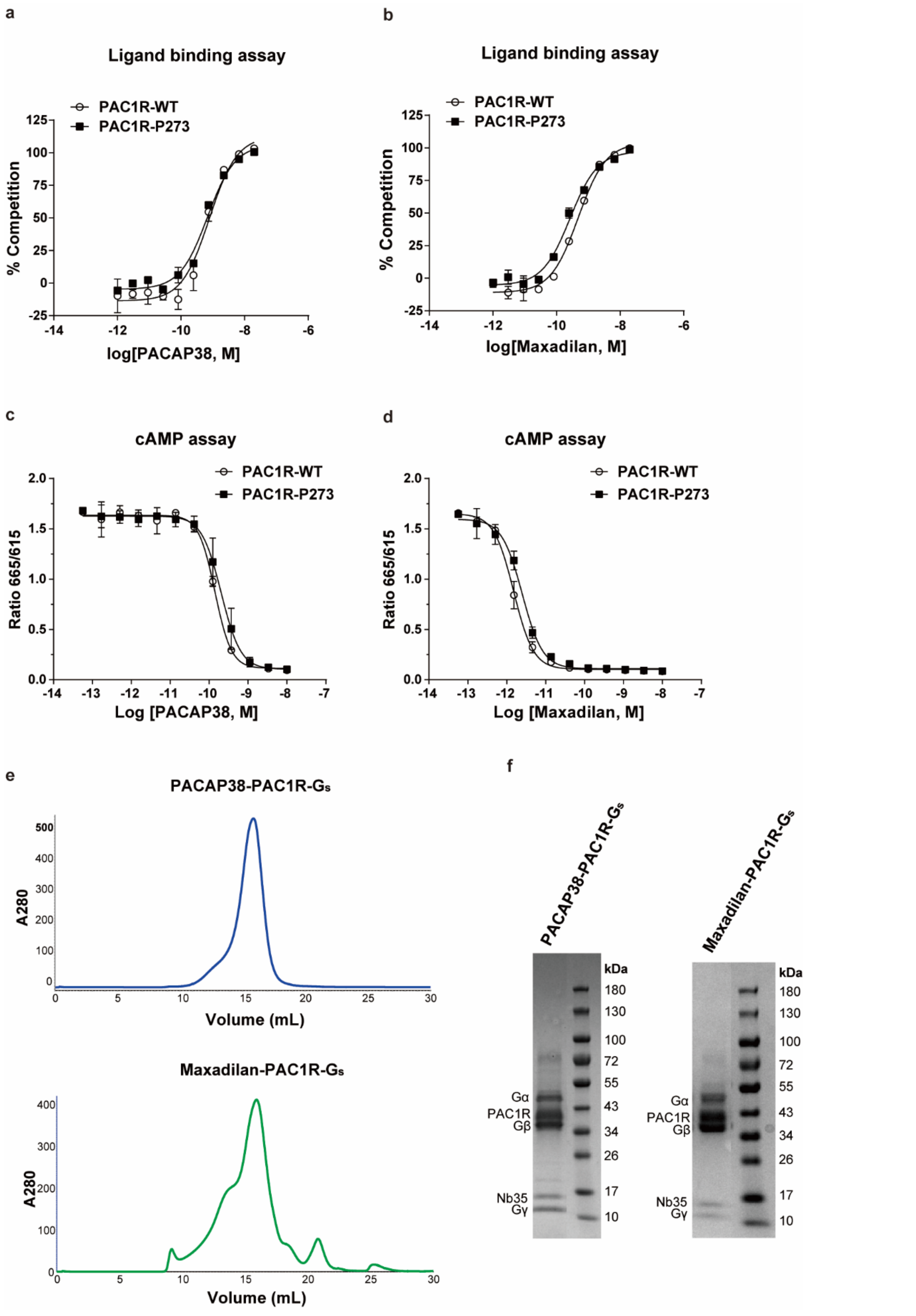

Fig. S2 PAC1R pharmacology, ligand binding and purification of PAC1R-G_s complex.

a, b Competition binding of PACAP38 (**a**) and maxadilan (**b**) to the WT PAC1R

(PAC1R-WT) and PAC1R used in cryo-EM studies (PAC1R-P273) in the presence of 0.2 nM ^{125}I- PACAP27, measured by the scintillation proximity assay. The plot shows data from a representative experiment with error bars representing s.e.m. from triplicate experiments. **c, d** Pharmacology of PAC1R-WT and PAC1R-P273, in PACAP38- (**c**) and maxadilan- (**b**) mediated functional cAMP assays performed in BacMam virus infected CHO-K1 cells. The plot shows data from n = 5 independent experiments with triplicate repeats with error bars representing s.e.m. **e** Final size-exclusion chromatography profiles of the PACAP38-PAC1R-G_s and maxadilan-PAC1R-G_s complexes. **f** SDS-PAGE and SimplyBlue (Invitrogen) staining of the size-exclusion chromatography peak fraction, demonstrating each component of the PACAP38-PAC1R-G_s and maxadilan-PAC1R-G_s complexes.
