# Supplementary material for: Cryo-EM structures of PAC1 receptor reveal ligand binding mechanism
Source: Cell Res. 2020 Feb 11;30(5):436–45. doi: 10.1038/s41422-020-0280-2 (PMC7196072; doi:10.1038/s41422-020-0280-2)
Supplement: Supplementary file 3 — Supplementary information, Fig. S3 [file 41422_2020_280_MOESM3_ESM.pdf]

Supplementary information, Figure S3

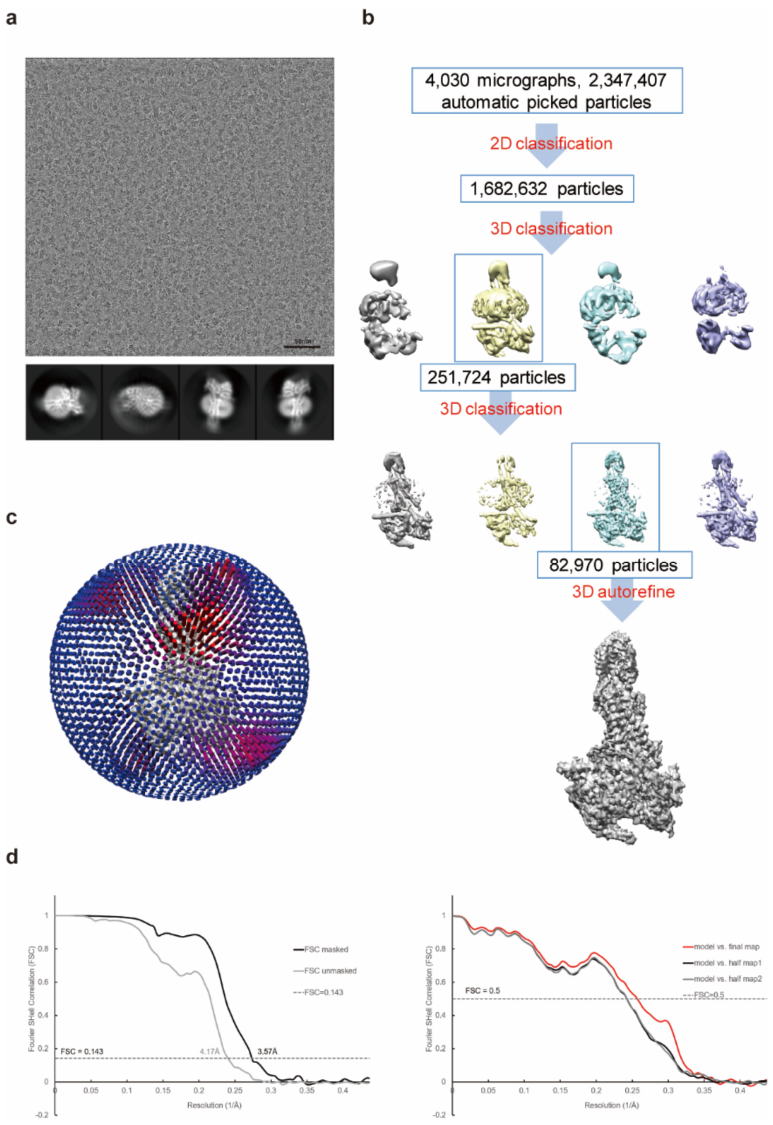

Fig. S3 Cryo-EM data processing for PACAP38-PAC1R-G_S. **a** Representative micrograph and RELION 2D class averages. **b** Workflow for map refinement and the final 3D cryo-EM map calculated in RELION after auto-refinement and map sharpening. **c** Angular distribution. **d** Left: gold standard Fourier shell correlation curve; the overall nominal resolution is 3.57 Å. Right: model fitting was evaluated by randomly displacing all atoms by 0.5 Å and refined against one cryo-EM half map. Fourier shell correlation curves were calculated between the final refined

model and the full map (red), the resulting model and the half map used for refinement (black); the resulting model and the other half map for cross validation (gray).
