# Supplementary material for: Cryo-EM structures of PAC1 receptor reveal ligand binding mechanism
Source: Cell Res. 2020 Feb 11;30(5):436–45. doi: 10.1038/s41422-020-0280-2 (PMC7196072; doi:10.1038/s41422-020-0280-2)
Supplement: Supplementary file 5 — Supplementary information, Fig. S5 [file 41422_2020_280_MOESM5_ESM.pdf]

Supplementary information, Figure S5

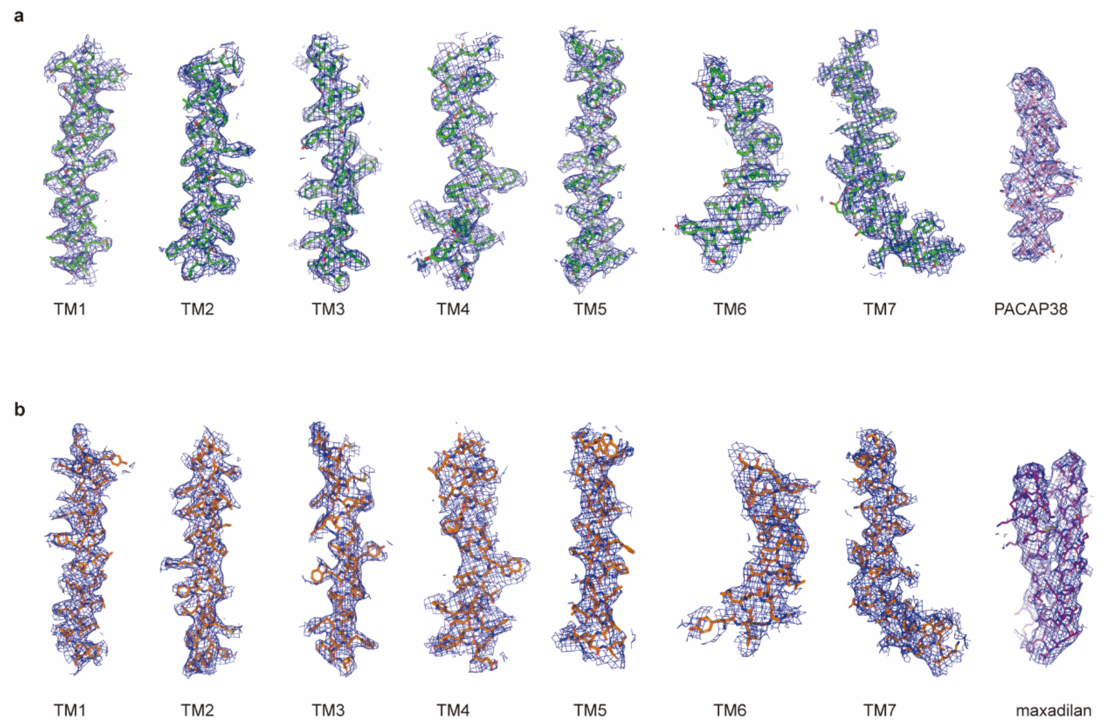

Fig. S5 Cryo-EM map quality. **a, b** Cryo-EM density map and model are shown for all seven transmembrane helices of the receptor in the PACAP38-PAC1R-G_s structure (**a**) and the maxadilan-PAC1R-G_s structure (**b**), the PACAP38 (**a**) and maxadilan (**b**) peptide.
