# Supplementary material for: Cryo-EM structures of PAC1 receptor reveal ligand binding mechanism
Source: Cell Res. 2020 Feb 11;30(5):436–45. doi: 10.1038/s41422-020-0280-2 (PMC7196072; doi:10.1038/s41422-020-0280-2)
Supplement: Supplementary file 6 — Supplementary information, Fig. S6 [file 41422_2020_280_MOESM6_ESM.pdf]

Supplementary information, Figure S6

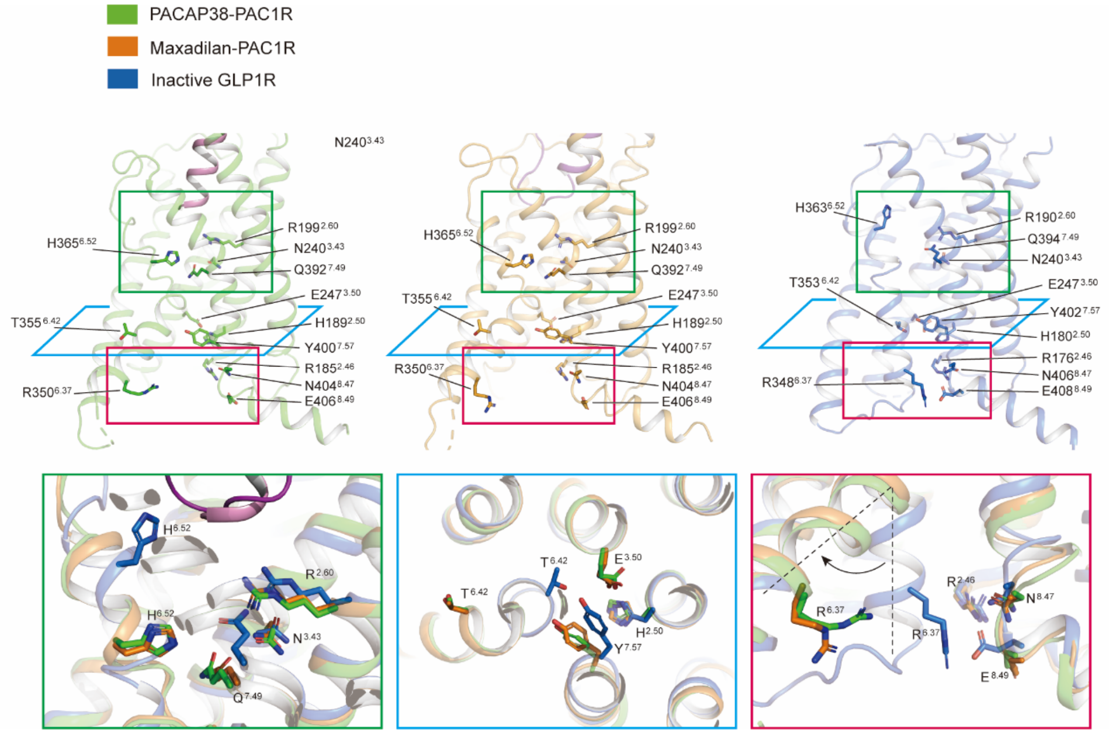

Fig. S6 Reorganization of the conserved polar networks upon ligand-induced PAC1R receptor activation. Inactive GLP1R (blue cartoon, PDB: 5VEX) is used to compare with the PACAP38 (green cartoon) and maxadilan (orange cartoon) induced active PAC1Rs. The close views show residue rearrangement at the conserved three layers of polar networks. Dash lines and arrows show the TM6 outward swing upon activation and G protein engagement.
