# Supplementary material for: Cryo-EM structures of PAC1 receptor reveal ligand binding mechanism
Source: Cell Res. 2020 Feb 11;30(5):436–45. doi: 10.1038/s41422-020-0280-2 (PMC7196072; doi:10.1038/s41422-020-0280-2)
Supplement: Supplementary file 7 — Supplementary information, Fig. S7 [file 41422_2020_280_MOESM7_ESM.pdf]

Supplementary information, Figure S7

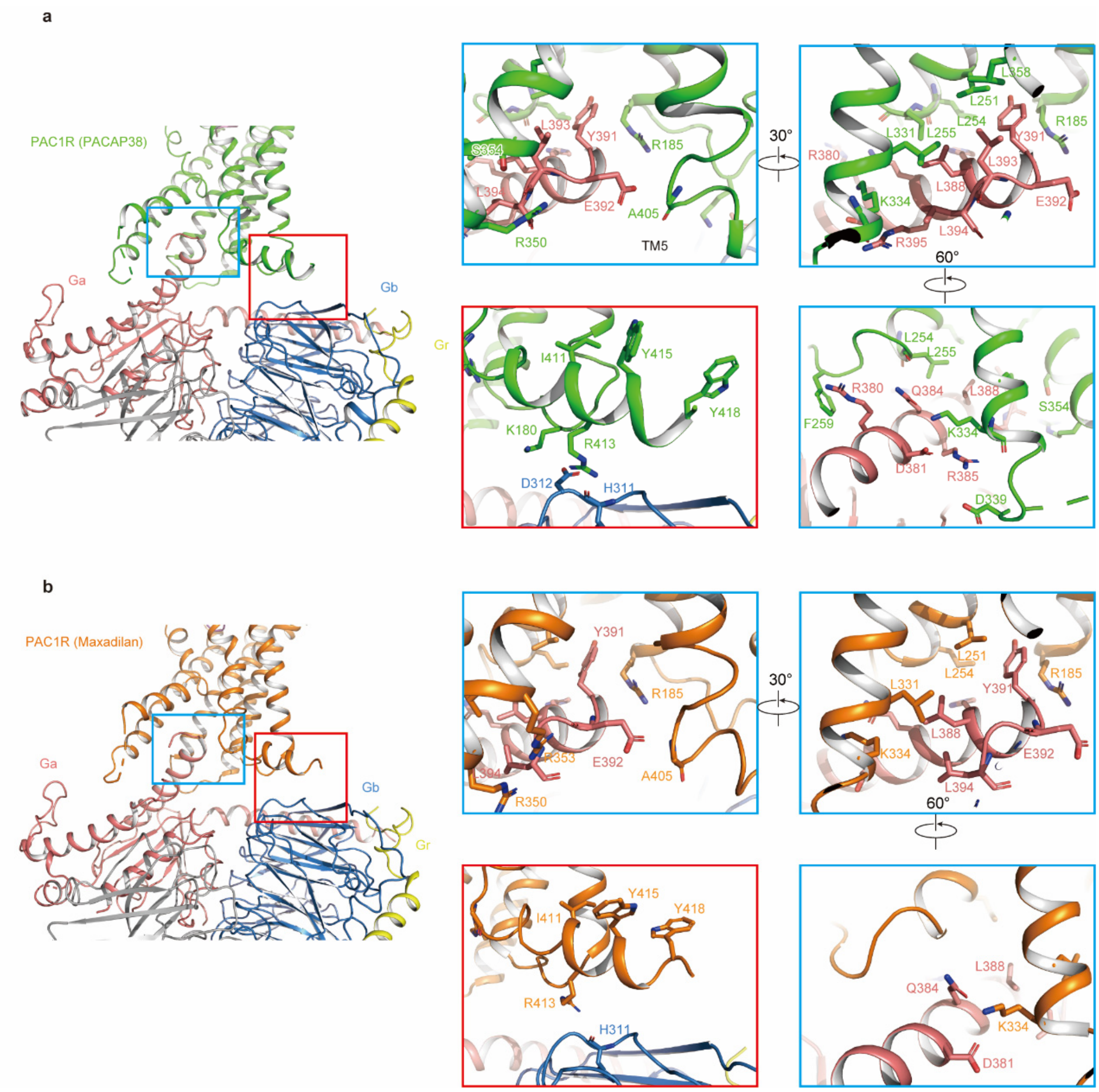

Fig. S7 PAC1R-G protein interactions. **a, b** Overall view and close views of PAC1R-G proteins interactions for PACAP38-PAC1R-Gs (**a**) and Maxadilan-PAC1R-Gs (**b**).
