# Supplementary material for: Cryo-EM structures of PAC1 receptor reveal ligand binding mechanism
Source: Cell Res. 2020 Feb 11;30(5):436–45. doi: 10.1038/s41422-020-0280-2 (PMC7196072; doi:10.1038/s41422-020-0280-2)
Supplement: Supplementary file 8 — Supplementary information, Fig. S8 [file 41422_2020_280_MOESM8_ESM.pdf]

Supplementary information, Figure S8

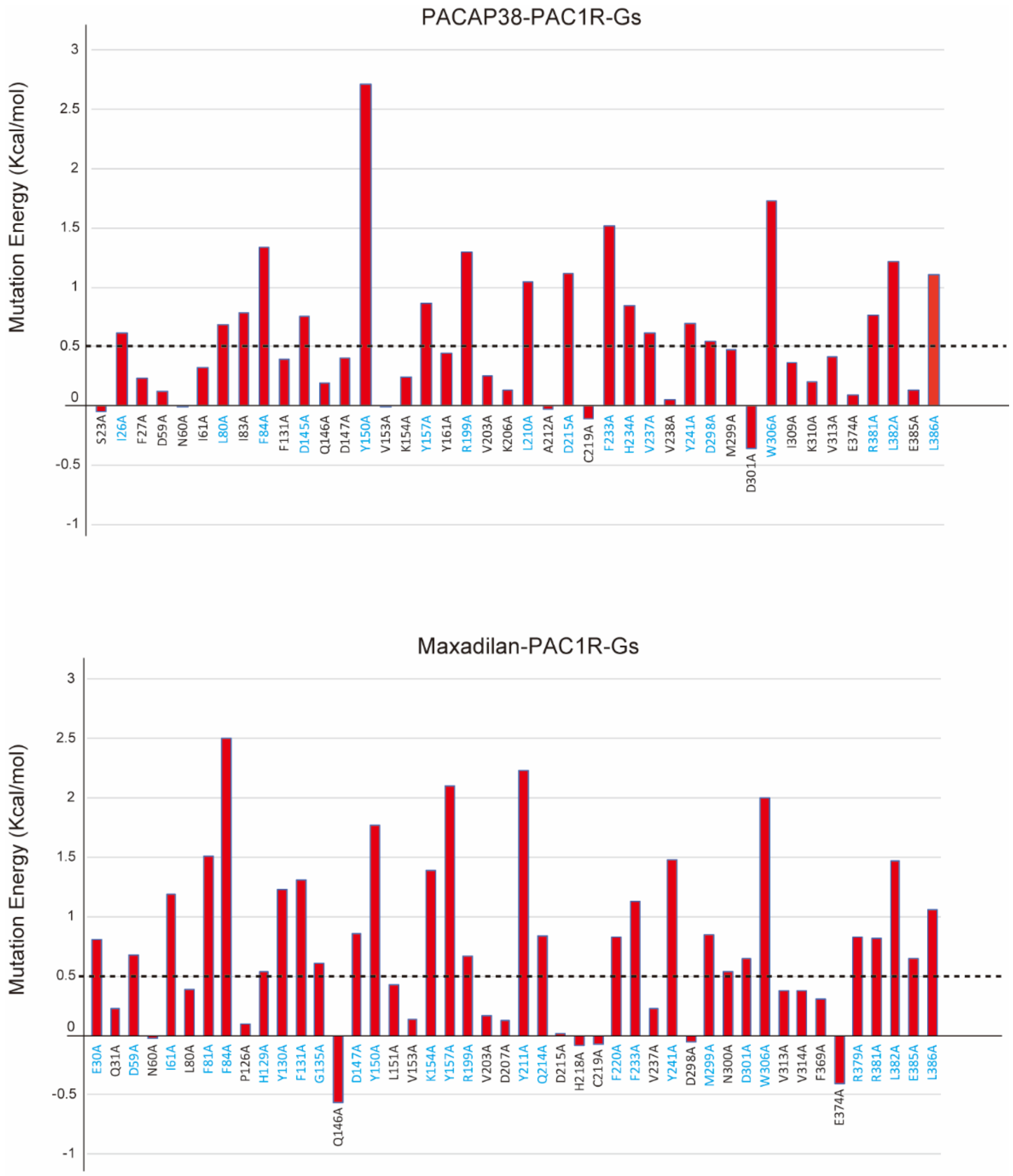

Fig. S8 Effect of the orthosteric site residues on ligand binding stability by virtual alanine scanning. The mutations with mutation energy higher than 0.5 Kcal/mol (black dash line) were selected for experimental validation.
