# Supplementary material for: Cryo-EM structures of PAC1 receptor reveal ligand binding mechanism
Source: Cell Res. 2020 Feb 11;30(5):436–45. doi: 10.1038/s41422-020-0280-2 (PMC7196072; doi:10.1038/s41422-020-0280-2)
Supplement: Supplementary file 9 — Supplementary information, Fig. S9 [file 41422_2020_280_MOESM9_ESM.pdf]

Supplementary information, Figure S9

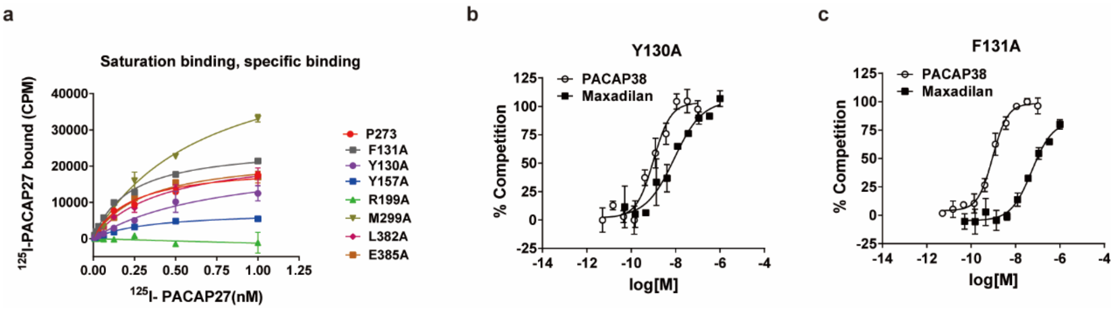

Fig. S9 Ligand binding assay of mutations affecting ligand-induced PAC1R activation. **a** Saturate binding of ^{125}I-PACAP27 to the mutants. **b, c** Competition binding of PACAP38 and maxadilan to Y130A (**b**) and F131A (**c**).
