# Supplementary material for: Cryo-EM structures of PAC1 receptor reveal ligand binding mechanism
Source: Cell Res. 2020 Feb 11;30(5):436–45. doi: 10.1038/s41422-020-0280-2 (PMC7196072; doi:10.1038/s41422-020-0280-2)
Supplement: Supplementary file 10 — Supplementary information, Fig. S10 [file 41422_2020_280_MOESM10_ESM.pdf]

Supplementary information, Figure S10

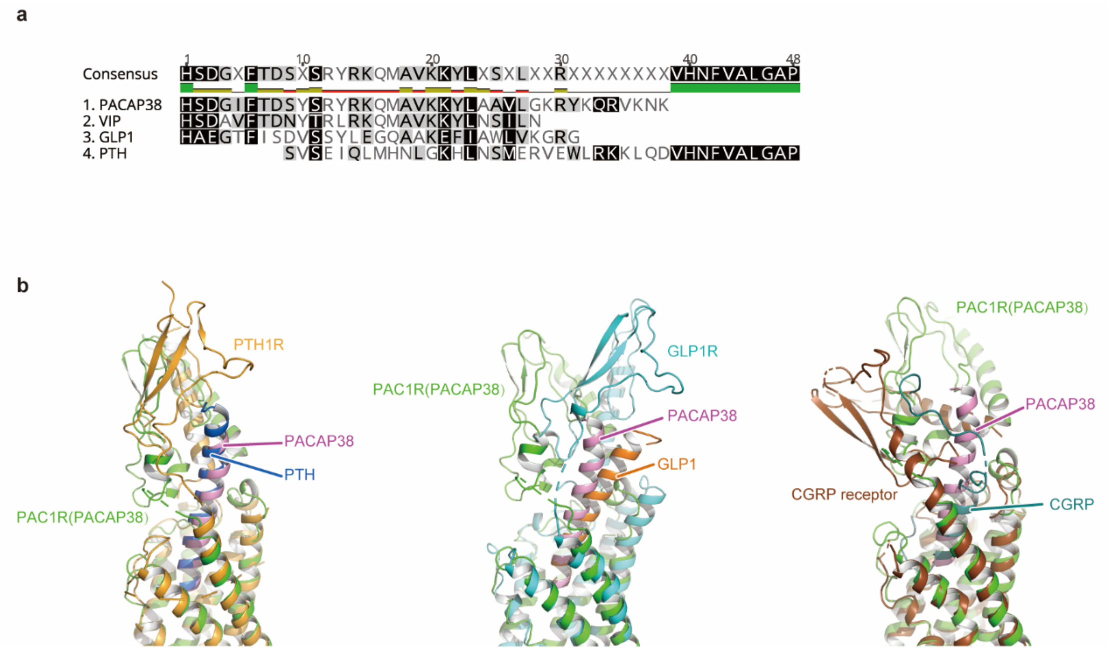

Fig. S10 Comparison of PACAP38-PAC1R-G$_s$ with other class B GPCR-G protein complex structures. **a** Sequence alignment for the ligand peptides of class B GPCRs (PACAP38, VIP, GLP1 and PTH). Highly conserved residues are highlighted in black and grey. **b** Structural superposition of PACAP38-PAC1R-G$_s$ to PTH-PTH1R (blue-gold cartoon), GLP1-GLP1R (orange-cyan cartoon) and CGRP-CGRP receptor (steel-brown cartoon), respectively, at the ligand binding region.
