# Supplementary material for: Cryo-EM structures of PAC1 receptor reveal ligand binding mechanism
Source: Cell Res. 2020 Feb 11;30(5):436–45. doi: 10.1038/s41422-020-0280-2 (PMC7196072; doi:10.1038/s41422-020-0280-2)
Supplement: Supplementary file 11 — Supplementary information, Table S1 [file 41422_2020_280_MOESM11_ESM.pdf]

Table S1 Data collection and refinement statistics

	PACAP38-PAC1R-G_s	Maxadilan-PAC1R-G_s
**Data collection**		
EM equipment	FEI Titan Krios	FEI Titan Krios
Voltage (kV)	300	300
Detector	Gatan K2 Summit	Gatan K2 Summit
Corrected Magnification	45829	45829
Pixel size (Å)	1.091	1.091
Electron dose ($e^-/Å^2$)	50	50
Exposure time (s)	5.6	5.6
Defocus range (μm)	1.5-2.5	1.5-2.5
Micrographs collected	4,519	3,382
Micrographs used	4,030	3,324
**Reconstruction**		
Software	RELION-3.0	
Total extracted particles	2,347,407	1,163,692
Number of final refined particles	82,970	58,451
Symmetry	C1	C1
Resolution unmasked (before post-processing, Å)	4.17	4.26
Resolution masked (after post-processing, Å)	3.57	3.63
Map sharpening B-factor ($Å^2$)	-100	-150
**Refinement**		
Number of atoms	9071	9092
RMSD		
Bond lengths (Å)	0.008	0.007
Bond angles (°)	0.947	0.934
Ramachandran Plot		
Favored (%)	92.84	93.82
Allowed (%)	7.16	6.18
Outlier (%)	0.00	0.00
Rotamer outliers (%)	0.31	0.20
MolProbity score	1.88	1.83
Clash score	7.69	7.73
