# Supplementary material for: Cryo-EM structures of PAC1 receptor reveal ligand binding mechanism
Source: Cell Res. 2020 Feb 11;30(5):436–45. doi: 10.1038/s41422-020-0280-2 (PMC7196072; doi:10.1038/s41422-020-0280-2)
Supplement: Supplementary file 12 — Supplementary information, Table S2 [file 41422_2020_280_MOESM12_ESM.pdf]

Table S2 Ligand-receptor interactions

PACAP38-PAC1R-Gs			Maxadilan-PAC1R-Gs		
**PACAP**	**PAC1R**	**Category**	**Maxadilan**	**PAC1R**	**Category**
H1	H234$^{3.37}$	Hydrophobic	R8	E30^{ECD}	Electrostatic
H1	V237$^{3.40}$	Hydrophobic	R8	N60^{ECD} (backbone)	Hydrogen Bond
S2	E385$^{7.42}$	Hydrogen Bond	H19	Q214^{ECL1} (backbone)	Hydrogen Bond
D3	Y161$^{1.47}$	Hydrogen Bond	H19	C219^{ECL1} (backbone)	Hydrogen Bond
D3	R199$^{2.60}$	Electrostatic; Hydrogen Bond	H19	F220^{ECL1}	Hydrophobic
F6	V153$^{1.39}$	Hydrophobic	S21	D147$^{1.33}$	Hydrogen Bond
S9	Y150$^{1.36}$	Hydrogen Bond	Q25	Y150$^{1.36}$	Hydrogen Bond
Y10	Y150$^{1.36}$	Hydrophobic	Q25	R379$^{7.36}$	Hydrogen Bond
Y10	L210$^{2.71}$	Hydrophobic	S27	K154$^{1.40}$	Hydrogen Bond
S11	D298^{ECL2}	Hydrogen Bond	V28	Y150$^{1.36}$	Hydrophobic
R12	M299^{ECL2}	Hydrophobic	V28	V153$^{1.39}$	Hydrophobic
R12	D301^{ECL2}	Electrostatic	A32	L386$^{7.43}$	Hydrophobic
Y13	D145$^{1.31}$	Hydrogen Bond	T33	Y241$^{3.44}$	Hydrogen Bond
Y13	Y150$^{1.36}$	Hydrophobic	F34	F369$^{6.56}$-A370$^{6.57}$ (backbone)	Hydrophobic
R14	L210$^{2.71}$ (backbone)	Hydrogen Bond	S36	N300^{ECL2}	Hydrogen Bond
K15	I83^{ECD}	Hydrophobic	M37 (backbone)	N300^{ECL2} (backbone)	Hydrogen Bond
K15	C219^{ECL1} (backbone)	Hydrogen Bond	T39 (backbone)	D301^{ECL2}	Hydrogen Bond
K15	M299^{ECL2}	Hydrophobic	K49	F136^{ECD}	Hydrophobic
Q16	I83^{ECD} (backbone)	Hydrogen Bond	K53	F136^{ECD} (backbone)	Hydrogen Bond
A18 (backbone)	D215^{ECL1}	Hydrogen Bond	K56	F131^{ECD}	Hydrophobic
A18	C219^{ECL1}	Hydrophobic	K56	F131^{ECD} (backbone)	Hydrogen Bond
V19	L80^{ECD}	Hydrophobic	F59	F131^{ECD}	Hydrophobic
V19	F84^{ECD}	Hydrophobic			
K21	D215^{ECL1}	Electrostatic			
V26	I61^{ECD}	Hydrophobic			
