# Supplementary material for: Cryo-EM structures of PAC1 receptor reveal ligand binding mechanism
Source: Cell Res. 2020 Feb 11;30(5):436–45. doi: 10.1038/s41422-020-0280-2 (PMC7196072; doi:10.1038/s41422-020-0280-2)
Supplement: Supplementary file 13 — Supplementary information, Table S3 [file 41422_2020_280_MOESM13_ESM.pdf]

Table S3 Receptor-G proteins interactions

PACAP38-PAC1R-Gs			
**PAC1R**	**G proteins**	**Category**	**Density map and structure comparation**
$R185^{2.46}$	$G_{\alpha s}$: Y391	Hydrophobic	Clear density
$L251^{3.54}$	$G_{\alpha s}$: Y391	Hydrophobic	Clear density
$L254^{3.57}$ (backbone)	$G_{\alpha s}$: H387	Hydrogen Bond	Clear density
$L254^{3.57}$	$G_{\alpha s}$: H387	Hydrophobic	Clear density
$L255^{3.58}$ (backbone)	$G_{\alpha s}$: Q384	Hydrogen Bond	Clear density; Distance 4.43 Å in Max structure
$L255^{3.58}$	$G_{\alpha s}$: L388	Hydrophobic	Clear density
$F259^{ICL2}$	$G_{\alpha s}$: V217	Hydrophobic	No density in Max structure
$F259^{ICL2}$	$G_{\alpha s}$: R380	Hydrophobic	No density in Max structure
$L331^{5.61}$	$G_{\alpha s}$: L388	Hydrophobic	Clear density
$L331^{5.61}$	$G_{\alpha s}$: L393	Hydrophobic	Distant in Max structure
$K334^{5.64}$	$G_{\alpha s}$: D381	Hydrogen Bond; Electrostatic	Clear density
$K334^{5.64}$	$G_{\alpha s}$: Q384	Hydrogen Bond	
$K334^{5.64}$ (backbone)	$G_{\alpha s}$: R385	Hydrogen Bond	Clear density; Distant in Max structure
$K334^{5.64}$	$G_{\alpha s}$: L394	Hydrophobic	Clear density; Orientation unfavorable in Max structure
$D339^{ICL3}$	$G_{\alpha s}$: R385	Electrostatic	Clear density; Distant in Max structure
$R350^{6.37}$	$G_{\alpha s}$: L394 (backbone)	Electrostatic	No density
$R350^{6.37}$	$G_{\alpha s}$: L394 (backbone)	Hydrogen Bond	No density
$S354^{6.41}$	$G_{\alpha s}$: L393 (backbone)	Hydrogen Bond	No density
$L358^{6.58}$	$G_{\alpha s}$: L393	Hydrophobic	Clear density; Distant in Max structure
$G405^{8.48}$ (backbone)	$G_{\alpha s}$: E392	Hydrogen Bond	Clear density
$K180^{ICL1}$	G_{β}: D312	Electrostatic	Clear density; Distant in Max structure
$R413^{8.56}$	G_{β}: H311 (backbone)	Hydrogen Bond	Clear density
**Maxadilan-PAC1R-Gs**			
$R185^{2.46}$	$G_{\alpha s}$: Y391	Electrostatic	Clear density
$R185^{2.46}$	$G_{\alpha s}$: Y391	Hydrophobic	Clear density
$L251^{3.54}$	$G_{\alpha s}$: Y391	Hydrophobic	Clear density
$L254^{3.57}$	$G_{\alpha s}$: H387	Hydrophobic	Clear density
$L331^{5.61}$	$G_{\alpha s}$: L388	Hydrophobic	Clear density
$K334^{5.64}$	$G_{\alpha s}$: D381	Electrostatic	Clear density
$K334^{5.64}$	$G_{\alpha s}$: Q384	Hydrogen Bond	Clear density
$R350^{6.37}$	$G_{\alpha s}$: L394 (backbone)	Electrostatic	No density
$R350^{6.37}$	$G_{\alpha s}$: L394 (backbone)	Hydrogen Bond	No density
$R353^{6.40}$	$G_{\alpha s}$: E392 (backbone)	Hydrogen Bond	Clear density; Distance 4.38 Å in Pac structure
$R353^{6.40}$	$G_{\alpha s}$: L394 (backbone)	Electrostatic	Clear density; Distance 7.1 Å in Pac structure
$G405^{8.48}$ (backbone)	$G_{\alpha s}$: E392	Hydrogen Bond	Clear density
$R413^{8.56}$	G_{β}: H311 (backbone)	Hydrogen Bond	Clear density
