# Supplementary material for: Cryo-EM structures of PAC1 receptor reveal ligand binding mechanism
Source: Cell Res. 2020 Feb 11;30(5):436–45. doi: 10.1038/s41422-020-0280-2 (PMC7196072; doi:10.1038/s41422-020-0280-2)
Supplement: Supplementary file 14 — Supplementary information, Table S4 [file 41422_2020_280_MOESM14_ESM.pdf]

Table S4 Mutagenesis of residues involved in the ligand binding pockets

	PACAP38			Maxadilan		
**Mutation**	pEC_{50}±SD*	Ratio to WT[#]	*p* value compared to WT[†]	pEC_{50}±SD	Ratio to WT[#]	*p* value compared to WT[†]
WT	9.74 ± 0.20	1.00		11.61 ± 0.20	1.00	
$P_{cryo\text{-}EM}$[1]	9.71 ± 0.21	1.09		11.51 ± 0.09	1.27	
Y130A	9.58 ± 0.51	1.45	0.719	10.08 ± 0.05	**34.04**	0.0002
F131A	10.0 ± 0.29	0.53	0.595	9.95 ± 0.25	**45.92**	0.0003
Y157A	9.21 ± 0.47	3.39	0.182	10.41 ± 0.13	**16.14**	0.0009
Y161A	8.36 ± 0.39	**24.04**	0.007	10.48 ± 0.20	**13.73**	0.0023
R199A	8.25 ± 0.36	**30.73**	0.005	10.92 ± 0.04	4.97	0.0041
K206A	8.49 ± 0.29	**17.77**	0.004	9.81 ± 0.11	**63.90**	0.0004
D207A	7.81 ± 0.29	**86.34**	0.001	10.27 ± 0.09	**21.98**	0.0012
M299A	9.27 ± 0.20	2.96	0.073	10.26 ± 0.09	**22.46**	0.0004
D301A	9.49 ± 0.12	1.79	0.229	10.41 ± 0.17	**15.99**	0.0014
W306A	8.54 ± 0.11	**15.84**	0.001	10.41 ± 0.17	**27.69**	0.0012
R381A	8.72 ± 0.19	**10.43**	0.002	10.42 ± 0.22	**15.54**	0.0009
L382A	9.24 ± 0.12	3.16	0.023	9.59 ± 0.06	**105.22**	0.0002
E385A	9.40 ± 0.27	2.22	0.213	9.71 ± 0.23	**80.03**	0.0004
	pEC_{50}[2]	Ratio to WT		pEC_{50}	Ratio to WT	
I26A	10.26	0.31		11.50	1.31	
E30A	10.10	0.44		11.53	1.22	
D59A	9.80	0.87		10.41	8.36	
I61A	9.92	0.67		11.36	1.80	
L80A	10.06	0.48		11.26	2.25	
I83A	9.95	0.62		11.45	1.45	
F84A	9.55	1.59		11.26	2.28	
F136A	10.23	0.33		11.63	0.96	
D145A	10.15	0.40		11.69	0.85	
D147A	10.05	0.50		11.71	0.81	
Y150A	8.98	5.86		10.85	5.82	
V153A	9.45	1.97		11.09	3.31	
K154A	9.72	1.07		11.46	1.44	
L210A	9.05	5.02		11.15	2.91	
Y211A	9.11	4.36		10.88	5.39	
Q214A	9.98	0.58		11.49	1.34	
D215A	9.70	1.13		11.46	1.42	
F233A	9.19	3.62		11.15	2.89	
H234A	8.86	7.70		10.96	4.53	
V237A	9.87	0.76		11.72	0.78	
Y241A	9.31	2.71		10.98	4.35	
D298A	9.56	1.55		11.28	2.17	
N300A	9.30	2.79		11.36	1.81	

R379A	9.58	1.47		11.28	2.17	
L386A	8.90	7.02		11.01	4.00	

*Potency displayed as an average of pEC_{50} ± standard deviation. Both the average and S.D. are calculated based 3 rounds of independent assays in 3 different days (N=3), and on each day, duplicates were run for each dose on dose response curves.

[#] Ratio to WT is calculated as EC_{50} of mutation to EC_{50} of WT.

[†] The best-fit value of pEC_{50} from 3 different assays was used to calculate *p* value in *T*-test, between WT and each mutation, with 2-tailed and equal variance (homoscedastic).

[1]PAC1R construct used in the cryo-EM studies

[2]Potency displayed as an average of pEC_{50} in one representative experiment.
